# *Porphyromonas Gingivalis* Load is Balanced by 0.20% Chlorhexidine Gel. A Randomized, Double-Blind, Controlled, Microbiological and Immunohistochemical Human Study

**DOI:** 10.3390/jcm9010284

**Published:** 2020-01-20

**Authors:** Simonetta D’Ercole, Gianmaria D’Addazio, Silvia Di Lodovico, Tonino Traini, Mara Di Giulio, Bruna Sinjari

**Affiliations:** 1 Department of Medical, Oral and Biotechnological Sciences, University “G. d’Annunzio” Chieti-Pescara, 66100 Chieti, Italy; simonetta.dercole@unich.it (S.D.); gianmariad@gmail.com (G.D.); t.traini@unich.it (T.T.); 2 Department of Pharmacy, University “G. d’Annunzio” Chieti-Pescara, 66100 Chieti, Italy; silvia.dilodovico@unich.it

**Keywords:** *Porphyromonas gingivalis*, dental implant, chlorhexidine, biofilm, TaqMan PCR, immunohistochemical analysis

## Abstract

Microbial contamination could compromise the stability of dental implants increasing the risk of inflammatory reactions in the surrounding soft tissues. In this human, randomized, double-blind, clinical study, the presence of *Porphyromonas gingivalis* on the healing abutment and the inflammatory infiltrate surrounding peri-implant soft tissues were investigated. Experiments were done in order to clarify the effect of 0.20% chlorhexidine (CHX) *versus* placebo, applied during each rehabilitation stage. Thirty patients (15 per group) were included. The load of adhering *P. gingivalis* on the healing screw were quantified by quantitative Polymerase Chain Reaction (qPCR) Taq-Man. Immunohistochemical analysis was carried out on the gingival biopsy. Moreover, clinical data were recorded. Analysis of variance and the Holm–Sidak test was used to evaluate differences between groups. The results showed a significant low presence of *P. gingivalis* load in healing abutments belonging to the 0.20% CHX group. Overall, the differences in terms of *P. gingivalis* DNA copy number between two groups were statistically significant (*p* < 0.01). All implants showed very low plaque and bleeding scores, but the placebo group appeared to have the highest expression of inflammation markers for T Lymphocytes, B Lymphocytes and macrophages Cluster definitions (CD3, CD20 and CD68). The use of 0.20% CHX could be recommended in all clinical procedures as it reduces significantly *P. gingivalis* load and host inflammatory response around implants.

## 1. Introduction

Dental implants are used by dentists for oral rehabilitation in partially and completely edentulous patients. The stability of implants is affected by an intimate contact between the marginal mucosa and implant abutment protecting the implant from undesired microbial colonization. Different factors can lead to dental implant failure [1,2,3]. This implant failure has been mainly attributed to peri-implantitis, a biological complication of oral implants, where periodontal pathogens cause sub-acute and chronic inflammation of the hard and soft tissues surrounding the implants [4,5]. Derks et al. reported an increase in peri-implantitis with frequencies ranging from 14% to 30% between 3 to 9 years of function [5]. Several authors demonstrated that peri-implantitis depends on the inability to maintain osseointegration. During oral disease, the production of bacterial metabolites can stimulate the increase of inflammatory markers that can cause the destruction of peri-implant tissues [6].

Bacterial colonization of the implant abutment interface begins directly after exposure to the oral environment. Microbiota has been identified 30 min after implant installation and within 2 weeks, with an established community similar to that found around natural teeth in the same mouth [7,8,9].

The long-term osseointegrated implants prognosis not only depends on the amount of the preformed biofilm, but also on the species embedded in the biofilm matrix and different microorganisms are involved in peri-implantitis [10]. The “red complex” bacteria, composed of *Porphyromonas gingivalis*, *Tannerella forsythia* and *Treponema denticola*, are accepted as strong etiological agents of peri-implantitis disease. In particular, among these microorganisms, Gram-negative *P. gingivalis* is the most strain isolated from many of the failing implants. Moreover, it is indicated as a “keystone pathogen”, because it plays an important role in the development and the progression of inflammatory disease, by altering a normally benign microbiota [11,12].

*P. gingivalis* produces virulence factors that facilitate colonization and induction of dysbiotic inflammatory responses such as capsules, fimbriae and proteases. [13]. In the colonization process, capsule shows a crucial role in the adhsion on human periodontal epithelial cells and in the co-aggregation with other periodonto pathogens. Moreover, it induces an important anti-phagocytic activity. Fimbriae, in addition to their adhesive function on oral surfaces, are belived to take part in the progression of periodontal inflammation. Proteases, such as gingipains, are involved in the colonization of periodontal pocket and implicated in the progression of periodontal disease together with the resistance to host defense mechanisms [14].

A new model of periodontal/peri-implant disease is emerging, the “polymicrobial synergy and dysbiosis” model [11,15,16,17]. This theory is based on the hypothesis that the inclusion of keystone pathogens, like *P. gingivalis*, shapes progressively a dysbiotic community causing a peri-implant disease [16]. According to this model, these kinds of pathogens shelterthe commensal community by immune subversion, and then transform the whole symbiotic microbiota into a more inflammophilic community. Thus, this will consist of large proportions of pathobionts capable to maintain dysbiosis and subsequent disease [16,18,19,20,21]. This hypothesis is supported by the evidence that *P. gingivalis*, instead of acting directly as a proinflammatory bacterium, has developed sophisticated strategies to evade or subvert components of the host immune system (e.g., Toll-like receptor and complement) [19]. Consequently, it has been hypothesized that *P. gingivalis* compromises innate immunity to alter the growth and development of the entire biofilm, triggering a destructive change in the normal host–microbial homeostatic interaction in the subgingival plaque [18,19,20,21]. The inflammation is the host response to bacterial insult and may explain the early bone resorption around implant connection [22]. Some authors have described the amount and type of peri-implant inflammatory infiltrate in healthy peri-implant tissues or in case of mucositis and peri-implantitis. They conclude that inflammatory infiltrates, composed of lymphocytes, macrophages, and plasma cells were present in the different sites in peri-implant soft tissues [23,24]. Gualini et al. in 2013 [23] described the peri-implant inflammatory infiltrate in patients with signs of mucositis and peri-implantitis using different clusters of differentiation (CD). Among these, CDs related to B and T lymphocytes are used as inflammatory markers. Similarly, Degidi et al. in 2006 [25] described the inflammatory infiltrate in soft tissues around healing screws through the use of T-Lymphocytes Cluster Differentiation (CD3), B-Lymphocytes Cluster Differentiation (CD20) and micro-vessel density (MVD). These studies have proven to be effective as a method to describe the inflammatory infiltrate in specific clinical conditions (zirconia healing screws or peri-implantitis) [23,25].

Several studies explored the origin of bacteria to cause peri-implantitis. The microgap present at the implant fixture-abutments interface seems to play a key role in the process of bacterial colonization. The microgap values are shown to range from 10 to 135 μm. This microgap can act as a reservoir for the bacteria resulting in a peri-implant inflammatory reaction and consequently peri-implant bone loss [26,27,28].

Nowadays, several strategies such as chlorhexidine (CHX) gels with different concentrations are used for the maintenance of soft tissue health and/or in the treatment of peri-implantitis. CHX is a local antiseptic usually applied after surgical procedures to reduce bacterial adhesion and biofilm formation on implant abutment surfaces [29,30,31,32,33]. The key role of CHX in inhibiting bacterial plaque formation has been widely confirmed [34,35]. This antiseptic remains the most effective antimicrobial agent used to reduce oral bacterial load affecting both Gram-positive and Gram-negative bacteria with bacteriostatic or bactericidal effects [36]. However, its benefits are limited because of its short-term application. At the best of authors’ knowledge this is the first clinical study that investigates, by means of microbiological and immunohistochemical analysis, the *P. gingivalis* colonization and the inflammatory infiltrate comparing CHX gel and placebo. This could give important information to clinicians on one of the factors that influence the early stages of peri-implant healing.

The aim of this paper, part of a randomized, controlled, double-blind, placebo/control study, was to investigate the effect of 0.20% CHX gel on the presence of *P. gingivalis* and on the inflammatory infiltrate surrounding peri-implant soft tissues. This research should clarify a possible relation between CHX gel and *P. gingivalis* colonization of implant abutment connection.

## 2. Experimental Section

### 2.1. Materials and Methods

#### 2.1.1. Study Design, Patient Selection and Randomization

**Figure 1:**
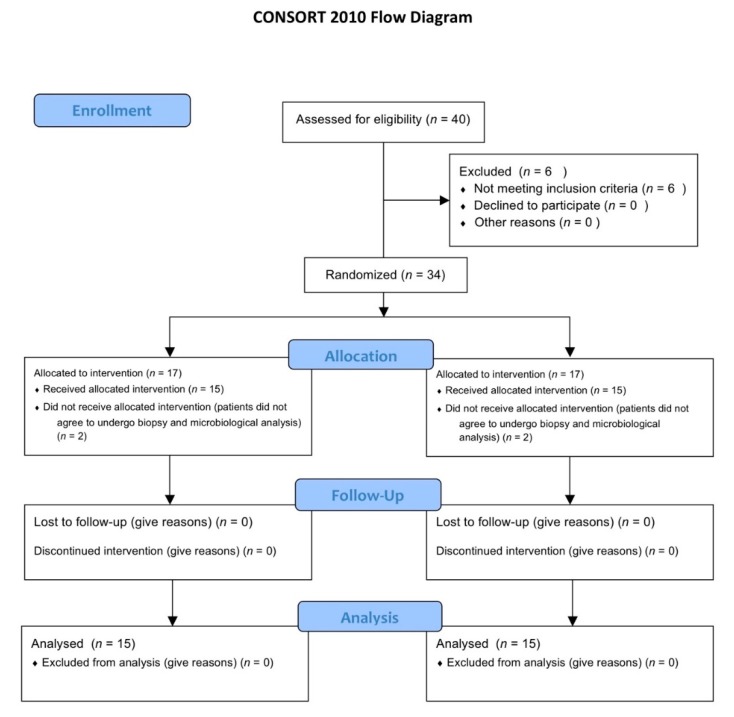
CONSORT 2010 flow diagram. Flow diagram of the progress through the phases of a parallel randomized trial of two groups.

This study is part of a prospective, randomized, controlled, double blind, clinical study designed according to the Declaration of Helsinki protocol. The allocation ratio was 1:1. The study was approved on 23/07/2015 by the Inter Institutional Ethics Committee of University of Chieti-Pescara, Chieti, Italy; committee report nr:14. All patients gave a written informed consent to the treatment and study recruiting. The trial was registered on clinicaltrials.gov with registration number NCT03431766 as previously described [32]. This article was written following the CONSORT statement for improving the quality of randomized controlled trials, RCTs (Figure 1) [37].

The stages of the study were organized as follows: T0, first surgical stage: implant placement, recording of clinical data and collection of soft tissue biopsies for immunohistochemical analysis (baseline); T1, second surgical stage after 8 weeks: recording of clinical data and collection of soft tissue biopsies for immunohistochemical analysis and insertion of healing abutment; T2, first prosthetic stage (implant impression) at 10 weeks: healing abutment collection for microbiological analysis (for more details see Table 1).

The primary outcome of this Randomized Clinical Trial (RCT) was to analyze the marginal bone resorption around dental implants as previously described in details [32]. Meanwhile, the objective of the present paper (the secondary outcome of the RCT) was to investigate by means of a quantitative analysis, the presence of *P. gingivalis* on the healing abutment when 0.20% CHX gel (Plak Gel; Polifarma Wellness Srl, Rome, Italy) and placebo gel (Placebo, Polifarma Wellness Srl, Rome, Italy) were applied during the first and second surgical stages. The two gels were perfectly alike in packaging, colour, and smell. Other secondary outcomes such as peri-implant soft tissues inflammation and gingival indexes were recorded. Plaque score (PS) and bleeding on probing (BOP) score were registered during each stage in every tooth (4 sides per tooth) [38]. The inflammatory infiltrate and the micro-vessel density (MVD) surrounding peri-implant soft tissues where also evaluated.

Forty patients were selected for implant placement at this RCT. Six were excluded because they did not meet the inclusion criteria. The inclusion criteria were: patients between 18 and 75 years, good systemic and oral health, need of single crown implant-supported restoration, at least six months of healing after tooth extraction, adequate dimension of the alveolar ridge and attached gingiva or keratinized tissue at the site selected. The exclusion criteria were: poor oral hygiene (PS and BOP more than 25%), active periodontal disease or other oral disorders, insufficient bone thickness for implant insertion, bone augmentation procedures, immediate loading protocols, uncontrolled diabetes mellitus, immune diseases, heavy smokers and bruxism. Four patients did not accept to receive biopsy and they were excluded from the microbiological and immunohistochemical analysis. Then, 30 patients (mean age 50.34 years; range 29–75 years) with good systemic and oral health condition, 14 women and 16 men were included for the immunohistochemical and microbiological analysis.

Patients were randomly divided into group A (placebo) and group B (0.20% CHX) as indicated by the randomization chart. The randomization was obtained using computer generated random numbers, centralized with sequentially sealed opaque envelopes provided by the study adviser. Patients were informed about the different gel but blinded to their allocation. The surgeon opened the sealed envelope containing the randomized group only after having inserted the implant.

The sample size of 30 patients was adequate to have a statistically significant difference between the two groups accordingly to a previous study [39].

#### 2.1.2. Surgical Treatment

**Figure 2:**
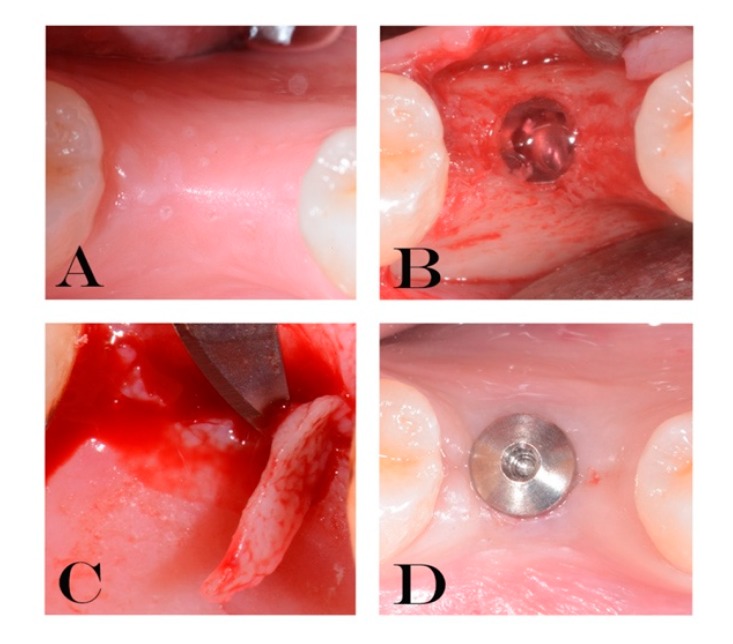
Explanatory images of the treatment performed (**A**–**D**). (**A**) residual crest. (**B**) gel inserted into the fixture (T0). (**C**) soft tissue biopsy (T1). (**D**) healing abutment placed (T1).

**Figure 3:**
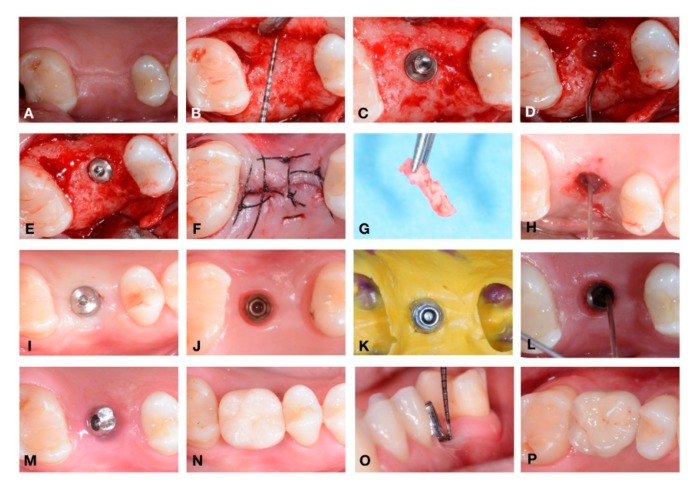
Detail of a complete case performed in this study: from implant placement to the delivery of permanent restoration. (**A**–**P**). (**A**) Residual crest before incision; (**B**) bone explosion with periodontal probe to underline the residual bone; (**C**) implant just positioned; (**D**) gel inserted into the fixture (T0) ; (**E**) insertion of cover screw; (**F**) suture; (**G**) soft tissue biopsy; (**H**) gel inserted into the fixture after the removal of cover screw; (**I**) healing abutment placed; (**J**) soft tissue before the impression; during this stage the healing abutment was collected for the microbiological analysis (T2) ; (**K**) impression; (**L**) gel inserted before the positioning of abutment; (**M**) definitive abutment location; (**N**) provisional crown; (**O**) probing around implant; (**P**) definitive ceramic restoration delivery.

During the first evaluation, all subjects were clinically examined: radiographs, plaque and bleeding scores were carried out for diagnostic evaluation; then the patients were scheduled for surgery procedures. Radiographs were performed to verify the presence of an adequate dimension of the alveolar ridge. All implants (Cortex classic, Shalomi, Israel) have been inserted at bone crestal level, by two skilled operators who followed a two-stage protocol and placed them according to the manufacturer’s instructions as previously described [32,40]. Implants inserted were all of 3.8 and 4.2 mm in diameter, with a mean diameter of 3.98 ± 0.20 mm and mean length of 11.07 ± 0.68 mm. In Figure 2 and Figure 3 it is possible to observe an explanatory case of the treatment performed. During the first surgical stage (T0) the gel was inserted into the fixture connection cave and closed with a cover screw. Also, gel was applied 2 times/day until suture removal after 7 days on the wound. Eight weeks later (T1) the second surgical stage was performed. Then, open a flap, removed the cover screw, inserted the gel again into the connection and inserted the healing screw. The healing abutment was tightened with an insertion torque of 15 Ncm. Gels were applied again, 2 times/day until suture removal after 7 days. At 10 weeks (T2), the healing abutments were collected to perform microbiological analysis.

**Figure 4:**
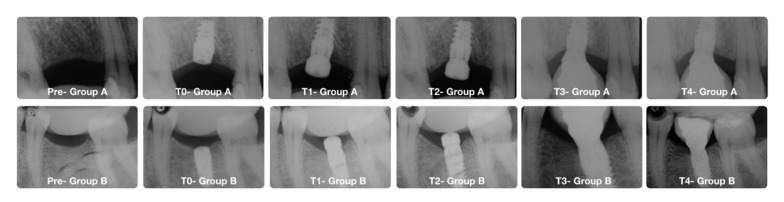
Radiographs from the two groups. In the upper images patient from Group A (control group) (clinical images are shown in Figure 3). In the lower images patient from Group B (test group). Pre (Image before the implant placement); T0 (after implant insertion); T1 (second surgical stage at 8 weeks); T2 (before the implant impression at 10 weeks); T3 (restoration’s delivery); T4 (1 year of follow-up).

Implant success rate was evaluated according to the clinical and radiographic criteria of Papaspyridakos et al. [41]: (1) absence of implant mobility; (2) absence of pain; (3) absence of recurrent peri-implant infection; and (4) absence of a continuous radiolucency around the implant. Data were collected to the specific patient’s case report forms (CRFs). In Figure 4 it is possible to see radiographs’ example from the two groups during the different time points.

The peri-implant and gingival indexes were recorded. Specifically, plaque score (PS) and bleeding on probing (BOP), were evaluated and recorded on four surfaces on each tooth at implant placement and at second surgical stage.

#### 2.1.3. Preparation of Total Genomic DNA

After 10 weeks of treatment with placebo gel (group A) or 0.20% CHX (group B), the healing abutment was collected to quantify the concentration of adhered *P. gingivalis*. Total genomic DNA was extracted from the titanium implant using the DNA Blood and Tissue Kit (Qiagen SpA, Milan, Italy). Briefly, each screw was resuspended in 300 μL enzymatic lysis buffer, and incubated for 1h at 37 °C. Then, 30 μL proteinase K and 200 μL buffer AL were added and incubated at 56 °C for 1 h. After addition of 200 μL ethanol, mixtures were transferred to DNeasy Mini spin columns (Qiagen) following the manufacturer’s protocol. Total genomic DNA was eluted in 100 μL and stored at −20 °C until use.

#### 2.1.4. TaqMan Polymerase Chain Reaction (PCR)

In-house TaqMan polymerase chain reactions (PCRs) were carried out in a total volume of 25 μL consisting of 12.5 μL Eurogentec PCR MasterMix No ROX (Eurogentec, RTQP2X- 03NR), containing buffer, dNTPs with dUTP, HotGold- Star DNA polymerase, 5 mM MgCl2, Uracil-N-glycosylase, and stabilizers), 300 nM of each forward and reverse primer 200 nM labelled probe, and 2.5 μL of template DNA. Quantification standards were amplicon carrying plasmids in three different concentrations (107, 105, and 102 copies/μL). Each sample was analyzed in triplicate. Thermal cycling, fluorescent data collection, and analysis were done with the CFX96 Touch Real Time System (BioRad Laboratories, Segrate, Milan, Italy). The cycling program was as follows: 1 cycle at 50 °C for 2 min and 1 cycle at 95 °C for 10 min followed by 40 cycles at 95 °C for 15 s and 60 °C for 40 s. The specific primers and probe used in this study were: Pg-F CCT ACG TGT ACG GAC AGA GCT ATA, Pg-R AGG ATC GCT CAG CGT AGC ATT, Pg-S FAM-TCG CCC GGG AAG AAC TTG TCT TCA-TAMRA, for the *P. gingivalis* Arg-gingipain (1 pro genome) [42,43,44].

For the construction of quantification standards, conventional PCR was conducted in 25 μL 1 × Biorad iQ-Supermix with 3 mM MgCl2 and 1 μM of each primer pair, under the following cycling conditions: 1 cycle at 95 °C for 5 min, followed by 40 cycles at 95 °C for 1 min, 52 °C for 1 min and 60 °C for 1 min. The amplicons were isolated using the High Pure PCR Purification Kit (Roche Diagnostics, Monza, Italy); briefly, 2.5 ng amplicon was ligated into 25 ng pGEM-T-vector by the Rapid DNA Ligation Kit (Roche Diagnostics, Rotkreuz, Schweiz, Switzerland) following the manufacturer’s instructions and subsequently propagated in *Escherichia coli* DH5α following Bizhang and colleagues’ methodology [43].

#### 2.1.5. Immunohistochemical Analysis

Soft-tissue biopsies were performed on the residual crest at the implant insertion (T0) and after two months of healing (second stage surgery) on the cover screw (T1) to analyze the peri-implant inflammatory infiltrate and the MVD in both groups. The size of the gingival biopsies averaged 2.1 mm (3.8–5.5 mm) in thickness and 3 mm in height. Thirty specimens (15 A and 15 B) were retrieved for evaluation during each surgical phase for a total amount of 60 specimens. All specimens were immediately fixed in 10% neutral buffered formalin and embedded in paraffin. Specifically, sections of three microns were obtained with a Leitz 1512 microtome and stained with hematoxylin-eosin (H&E). The immuno-histochemical staining of CD20, CD3, CD68, CD31 (OriGene Technologies, Inc., Rockville, MD, USA) was performed using the Streptavidine-Biotine-Peroxidase (strep-ABC) method as previously described [45]. Same sections were cut and mounted on poly-L-lysine-coated slides. Paraffin sections were de-waxed by xylene, rehydrated and finally washed in phosphate-buffered saline (PBS, pH 7.4) for 10 min. In order to unmask the antigens, a microwave oven and 2.1% content of citric acid was used related to the antibodies CD20, CD3, CD68, CD31. Eventually the process was optimized by using automatic staining (Optimax, BioGenex, San Ramon, CA, USA). Sections were incubated with primary antibody for 30 min at room temperature. Slides were rinsed in buffer, and immuno-reaction was completed with the Strep-ABC-Peroxidase method, applying the “Super sensitive immuno-detection” kit by BioGenex (San Ramon, CA, USA) utilizing a multi-link as a secondary biotinilated antibody. After incubation with a chromogen employing “liquid DAB substrate pack” (BioGenex, San Ramon, CA, USA), the specimens were counterstained with Mayer’s hematoxyline and coverslipped. Five random fields at higher magnification (20×) were chosen from each sample. Images at higher magnification were captured using a dedicated software (Leica QWIN, version 3.0, Leica Microsystems Imaging Solutions Ltd, Coldhams Lane, Cambridge, UK) connected to a light microscope (Leica DMR, Leica Microsystems Imaging Solutions Ltd, Coldhams Lane, Cambridge, UK). The software was calibrated to detect automatically the positivity of each marker and quantify the area; this allowed calculating, for each field, the percentage of positiveness of CD3, CD20, CD68.

A different procedure was applied to examine CD31. In order to calculate the density and the number of vessels each single field was counted (200×) taking care of excluding false positives. Each field had an area of 239850.5032-micron^2^, corresponding to approximately 0.2399 mm^2^. Each sample was analyzed on 5 random fields with a total area of 1.1 mm^2^. The individual shapes of the vessels were underlined in order to prevent repetition or omission. The values were expressed as numbers of micro-vessels for 0.2399 mm^2^ soft tissue (MVD).

#### 2.1.6. Statistical Analysis

In this clinical trial, sample size was calculated following the primary outcome that was the marginal bone loss (MBL). Data of the MBL was previously published [32]. Specifically, a sample size of 15 patients per group was calculated to have at the follow-up a minimum difference of MBL between the two groups of −0.55 mm with an expected standard deviation of 0.5 mm. The value of α was determined at 0.05 while the power of the test was 0.80. For the calculation, the Pass software (version 13, NCSS, LLC., East Kaysville, UT, USA.) was used and specifically the two-sample *t*-tests taking equal variance.

Statistical analysis was performed using SPSS v.26 (IBM Corp., Armonk, NY, USA) and Excel (Microsoft, Redmond, WA, USA) softwares. The statistical tests chosen were predetermined by the study protocol. Patients were considered for statistical evaluation. Data were presented by means of standard deviations (SD). Analysis of variance (Student’s *t*-test) was used to evaluate differences between single markers mean values. The Holm–Sidak test was used to evaluate different markers among groups. Significance was set to *p* = 0.05.

## 3. Results

All patients appeared clinically healthy in both surgical phases (T0, first surgical stage, implant insertion and T1, second surgical stage). No clinical signs of inflammation or infection were detected during all surgical phases and all implants were osseointegrated with a survival rate of 100%. Moreover, no dropouts occurred during the whole study. All registered indices (plaque score and bleeding score) demonstrated that in both groups (A, placebo group and B, 0.20% CHX group) were less than 25% during the two surgical phases with a slight increase at the second surgical stage in both groups, but without statistically significant differences (Table 2).

**Figure 5:**
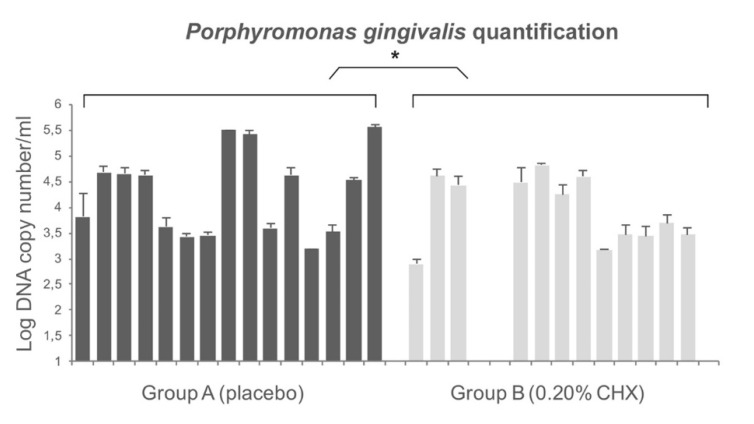
Quantification of *P. gingivalis* on healing abutment by TaqMan Polymerase Chain Reaction (PCR). The differences in terms of colony forming units (CFU) between two groups were statistically significant as reported by * in the picture.

The total concentration of *P. gingivalis* adhering to healing abutment was quantified by TaqMan PCR comparing to the standards curve. The efficiency of the TaqMan PCR ranged from 94% to 99% and the coefficient of determination ranged from 0.98 to 0.99 (ideal value = 1.00). The quantification of *P. gingivalis* with TaqMan PCR shows the presence of this bacterium in all healing screws except for three samples belonging to the 0.20% CHX group (group B). The concentration of *P. gingivalis* for group A ranged from 1.5 × 103 CFU/mL to 3.5 × 105 DNA copy number/mL and the values for group B ranged from 0 to 6.3 × 104 DNA copy number/mL. The results showed a significant low concentration of *P. gingivalis* in healing abutments belonging to the 0.20% CHX group. Overall, the differences in terms of DNA copy number between the two groups were statistically significant (*p* < 0.01). Figure 5 shows the detailed values.

**Figure 6:**
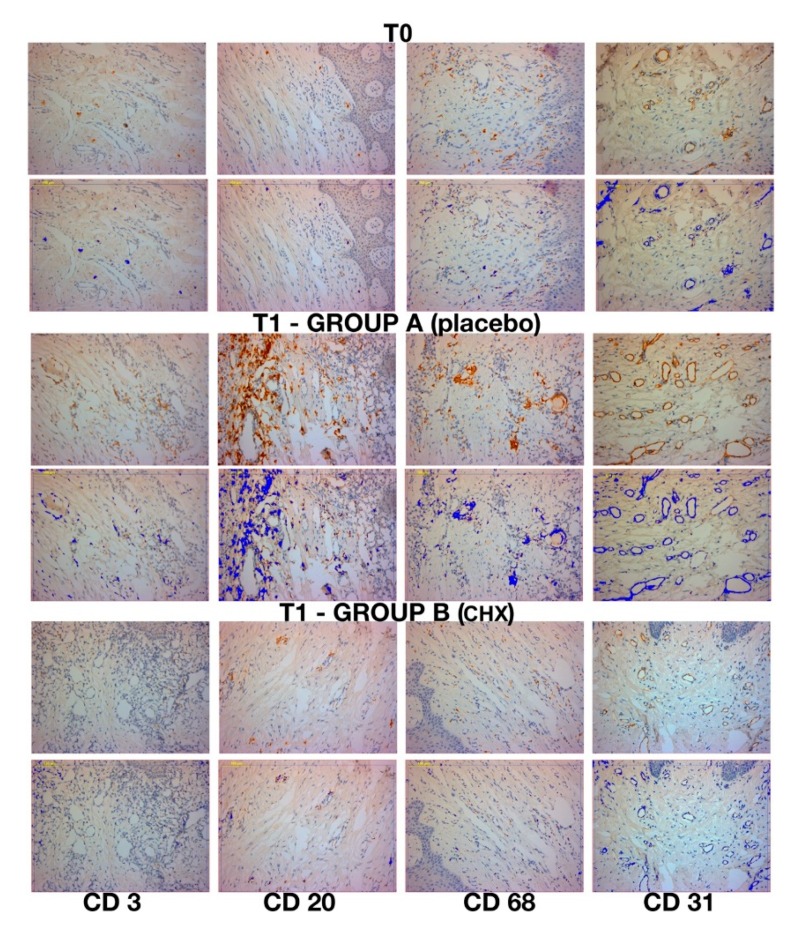
Immunohistochemical expression at T0, and T1 in group A and B (second surgical stage). Each of these images are in duplicates as one represent the immunohistochemical stain (strep-ABC 20× magnification) meanwhile the duplicate represents the image software analysis (and software analysis 20× magnification). In the first column CD3 expression (strep-ABC 20× magnification and software analysis 20× magnification). In the second column CD20 expression (strep-ABC 20× magnification and software analysis 20× magnification) are shown. In the third column CD68 expression (strep-ABC 20× magnification and software analysis 20× magnification) are shown. In the fourth column CD31 expression (strep-ABC 20× magnification and software analysis 20× magnification).

Although clinically healthy, inflammatory cellular elements were present in all samples at T0 (first surgical stage, implant insertion) and T1 (second surgical stage) (Figure 6). The inflammatory infiltrate was represented by T lymphocytes (CD3), B lymphocytes (CD20), and macrophages (CD68). At T0, very low inflammation level was shown in the pre-surgical stage of every sample. Specifically, the values for group A at T0 were: CD3 = 2.76% ± 1.3, CD20 = 3.36% ± 1.45 and CD68 = 2.55% ± 0.67. The values for group B were: CD3 = 2.56% ± 1.24, CD20 = 3.12% ± 1.56, CD68 = 2.61% ± 1.1. The micro-vessel density (MDV) was 19.4 ± 2.7 and 18.79 ± 2.68 per mm2 for group A and B, respectively. No statistically significant difference between groups was found (*p* > 0.05).

Group A samples showed at T1 variable inflammation levels and vessels density. These samples appeared to have the highest expression of inflammation markers for CD3, CD20 and CD68, the values shown were: CD3 = 7.02% ± 1.4, CD20 = 13.78% ± 2.0 and CD68 = 5.41% ± 0.8. Meanwhile, the samples for group B showed a lower percentage of inflammatory infiltrate for CD3, CD20 and CD68, of 2.91% ± 1.1, 4.96% ± 1.9, 2.53% ± 0.9, respectively. Moreover, the MDV for both groups were 32.6 ± 3.4 per mm^2^ and 21.88 ± 3.0 per mm^2^ for group A and B, respectively. In Figure 6 is shown the expression of CD31 for both groups used to calculate the MDV. The analysis of data revealed statistical difference between the two groups (*p* < 0.001) (Table 3). Moreover, a higher number of T lymphocytes (CD3+) and of B lymphocytes (CD20+) were observed in group A (placebo). Table 3 showed a statistical difference between the single markers expression for the CD3, CD20, CD68 and CD31. A statistical significance was shown in group A between T0 and T1, meanwhile no statistical differences was shown for group B.

## 4. Discussion

This study aimed to verify the effect of placing 0.20% CHX gel in the early stages of healing after implant insertion on both the inflammatory infiltrate in peri-implant soft tissues and on the *P. gingivalis* presence on the healing screws. The results obtained encourage the use of CHX within the implant connection, during post-operative healing and during the second surgical stage since it demonstrated a significant reduction of *P. gingivalis* load and inflammatory infiltrate. This treatment in respect of that of the placebo also guaranteed excellent levels of tissue healing based on clinical parameters.

It is well known that, to preserve crestal bone loss and guarantee a clinical healthy state around dental implants, it is essential to ensure the decontamination of the implant-healing abutment interface and to use antiseptics during the surgical and prosthetic phases for reducing the degree of contamination [25,46]. Several studies investigated implant materials, surfaces, roughness, healing caps and abutments [40,47,48,49], but, to the best authors knowledge, this is the first clinical study that investigates, by means of microbiological and immunohistochemical analysis, the *P. gingivalis* colonization and the inflammatory infiltrate using CHX gel from first to second surgical stage and in prosthetic stage. Chlorhexidine is mostly used in chemical plaque control and is effective in decreasing plaque accumulation for its bacteriostatic and bactericidal activities against both Gram-positive and Gram-negative bacteria. Many authors described the use of CHX as a support treatment for peri-implantitis [46,50,51,52] although, in some cases, its effect seems questionable. In particular, Carcuac et al. [53] studied the role of systemic and local medications in the treatment of peri-implantitis concluding that the use of CHX did not improve the outcomes. These data are only in apparent contrast with our results since the authors used CHX in surgical phase, and not in both surgical and prosthetic phases as we did. Podhorsky et al. assessed that the contamination of the internal implant part was drastically reduced by using CHX [53]. In support of this, other authors investigated the presence of bacteria within the connection, demonstrating a positive effect of CHX up to six months after loading [29,30].

In our study, *P. gingivalis* load is balanced by the use of CHX gel, in fact, bacterial count was statistically higher in placebo group than in treated samples. Bacterial colonization can be caused either by contamination of the microgap or as a result of contamination during implant placement. Studies showed an increase in bacterial colonization of the interface over time, demonstrating the key role of the microgap [26,27,28,54,55,56,57]. Canullo et al. demonstrated an association between peri-implantitis progression and peri and/or internal implant bacterial contamination. They reported that the difference in bacterial counts of the Red and Orange Complex was more pronounced within the interface compared with peri-implant colonization [57,58]. It was clear that the internal component of the fixture worked as a bacterial reservoir.

The TaqMan PCR results demonstrated that *P. gingivalis* was present in all healing screws except for three samples belonging to the 0.20% CHX group. However, as also reported by others [48], this bacterium is detected both in healthy and peri-implantitis sites. Plaque score and bleeding score were very similar between groups but *P. gingivalis* loads differ between it. The reason should be that the plaque index gives an estimation of the quantity of the plaque around implants but it does not give a qualitative one. In fact, these results suggest that in the sites where the 0.20% CHX gel has been inserted the quality (less *P. gingivalis* pg load has been observed) of the plaque is different from the placebo one, although the same plaque index. *Porphyromonas gingivalis* load represents a crucial element in the unbalance of the commensal oral microbiota. Moreover, low concentrations of the microorganism are not sufficient to induce peri-implantitis. In fact, as pathobiont, it is a natural member of oral microbiota [59] but, as reported by Al-Ahmad et al. [60], its growing concentration combined with high levels of the Red Complex could cause the shift of the microbiota from a healthy status to a diseased inducing peri-implantitis. The limitation of our study includes the evaluation of the CHX 0.20 gel effect on the *P. gingivalis* presence although it is not the only responsible for peri-implantitis. In fact, as reported by other authors, peri-implantitis has been related to the colonization of various microorganisms such as *P. gingivalis*, *Prevotella intermedia* and *Aggregatibacter actinomycetemcomitans*, *Fusobacterium* spp., and *Staphylococcus* spp. [5]. Among these strains, *P. gingivalis* is recognized as most closely related to the development of subgingival and periodontal infections including peri-implantitis [11]. Although, the link between bacterial load and peri-implantitis is very important and results regarding this subjects could provide answers about this complex relationship, another limitation of the present study is that it is part of a RCT where the primary outcome was the marginal bone loss.

The important role of *P. gingivalis* in the oral microbiota balancing is supported by a recent study in the mouse model through the *P. gingivalis* keystone hypothesis [12]. The authors reported that a very low colonization levels of *P. gingivalis* (<0.01% of the total bacterial count) induced periodontal disease and significant alterations in the number and organization of the oral community commensal bacteria. These changes precede the onset of inflammatory bone destruction and, therefore, lead to the consideration that dysbiosis is probably the cause of the disease [12].

Peri-implant bacterial colonization cause an inflammatory reaction in the peri-implant soft tissue similar to gingivitis in the natural tooth [61]. Ericsson et al. showed that this reaction was an attempt by the host defenses to react to peri-implant bacterial biofilm, also present in sites under strict plaque control [22]. It is important to remember that in this study all implants were perfectly osseointegrated, with plaque and bleeding scores less than 25%. The clinical health status of the implants was acceptable in all samples. No cases of suppuration or infections occurred at the second surgical stage. The choice to select patients with adequate bone level (width >5 mm) and good amount of keratinized gingiva probably allowed to obtain an adequate healing. In all specimens, inflammatory infiltrate levels obtained by immunohistochemical analysis were acceptable, although with significant differences. In particular, at implant insertion, no significant statistical difference was found between the groups. The use of 0.20% CHX gel, applied during each surgical and prosthetic stage, demonstrated statistically significant differences in respect to placebo group. In fact, all placebo samples showed an important percentage of inflammatory infiltrate mainly consisting of T lymphocytes (CD3+) and B lymphocytes (CD20+). In addition, a large number of vessels, measured as expression of the CD31 marker, were present in placebo samples. Thus, these results can be explained as a higher rate of inflammatory-related repairing processes in these samples. In our samples, the presence of bacteria within the interface probably correlates to the presence of peri-implant inflammatory cells caused by the micro-pumping of bacteria and bacterial products to the surrounding implant tissues through the abutment microgap. Therefore, these results demonstrate how the increase of *P. gingivalis* can cause an increase of the infection-related inflammatory infiltrate linked with the bacterial load. This increase in bacterial load can be controlled with the use of CHX gel. The control of oral hygiene is certainly a fundamental aspect but associated with the use of correct aids in the post-operative period, and in subsequent stages it guarantees a control of the relevant infection. As already mentioned, the results referred to the first stages of rehabilitation where the gel was used at each connection and disconnection of the components. Further studies could provide information on this by removing the prosthesis after a long follow-up period. Mainly for ethical reasons, this is not possible and could only be performed in conjunction with removals for other reasons (mechanical or prosthetic). In conclusion, the results of this study show that the use of CHX considerably reduces *P. gingivalis* presence, peri-implant inflammation and improve the clinical outcomes of implant-supported restoration. Different in vitro studies showed how CHX was cytotoxic for fibroblast cells, myoblast, osteoblast, cell viability and migration [62]. However, its in vitro cytotoxicity is higher for concentrations close to 2%. In this study, a concentration of 0.20% was used, inside the implant fixture as shown in Figure 2 and Figure 3. For these reasons, the aforementioned cytotoxicity can be negligible. In the literature, there are no other real clinical side effects reported for CHX, excluding a partial and reversible alteration of the colour of the teeth [63]. However, this adverse effect is negligible in treatment with topical gel, as used in the present study.

## 5. Conclusions

Since, there are no specific guidelines regarding the time and method of CHX usage, our methodology lays the foundation or suggests the use of 0.20% CHX gel in all the clinical phases to reduce the *P. gingivalis* load and consequently to improve the soft and hard peri-implant tissues healing. The present paper, emphasises the crucial role of our suggested specific rigorous procedure based on the use of 0.20% CHX inside the implants and in all evaluated stages.

## Figures and Tables

**Table 1:** Timeline of stages performed.

Timeline Stages			
Stage	Time Point	Clinical Procedures	Experimental Procedure
T0	0 weeks	Implant insertion	Soft-tissue biopsy
T1	8 weeks	Second surgical stage	Soft-tissue biopsy
T2	10 weeks	Implant impression	collection of healing abutment for microbiological analysis

**Table 2:** Bleeding on probing (BOP) and plaque score at implant placement and at second surgical stage.

Clinical indexes.	Group A (*n* = 15) (PLACEBO).	Group B (*n* = 15) (CHX)
BOP mean at implant placement	9.2%	8.15%
BOP mean at second surgical stage	11.32%	12.21%
Plaque score at implant placement	17.45%	17.32%
Plaque score at second surgical stage	19.34%	18.45%

**Table 3:** Inflammatory infiltrate at second surgical stage (T1) expressed as mean and standard deviation and analysis of Variance. Statistical significant differences between single markers were found (*p* < 0.001). Group A: placebo samples. Group B: Chlorhexidine (CHX) samples.

Markers	CD 3	CD 20	CD 68	CD 31
***GROUP A (PLACEBO)***				
No.	15	15	15	15
Mean (SD)	7.02% ± 1.4	13.78% ± 2.0	5.41% ± 0.8	32.61 ± 3.4
Range	(4.086–10.264)	(10.74–17.708)	(4.042–6.97)	(22–36)
SEM	0.375	0.527	0.225	0.881
***GROUP B (CHX)***				
No.	15	15	15	15
Mean (SD)	2.91% ± 1.1	4.96% ± 1.9	2.53% ± 0.9	21.8 ± 3.0
Range	(1.36–4.55)	(2.636–9.778)	(0.824–4.01)	(17–27)
SEM	0.309	0.508	0.225	0.786
Difference	4.111	8.825	2.877	10.733
T (with 28 degree of freedom) (*p* < 0.001)	8.455	12.056	8.455	9.093
95% confidence interval for difference of mean	3.115–5.107	7.326–10.324	2.180–3.574	8.315–13.151
*p* value	*p* < 0.001	*p* < 0.001	*p* < 0.001	*p* < 0.001

SD: standard deviation.
